# The history of optic chiasm from antiquity to the twentieth century

**DOI:** 10.1007/s00381-017-3564-1

**Published:** 2017-08-14

**Authors:** Claudia Florida Costea, Şerban Turliuc, Cătălin Buzdugă, Andrei Ionuţ Cucu, Gabriela Florenţa Dumitrescu, Anca Sava, Mihaela Dana Turliuc

**Affiliations:** 1 Department of Ophthalmology, Gr. T. Popa University of Medicine, Iasi, Romania; 2 Nicolae Oblu Emergency Clinical Hospital, Iasi, Romania; 3 Department of Psychiatry, Gr. T. Popa University of Medicine, 16 University Street, Iasi, Romania; 4 Department of Endocrinology, Gr. T. Popa University of Medicine, Iasi, Romania; 5 Department of Anatomy, Gr. T. Popa University of Medicine, Iasi, Romania; 6 Department of Neurosurgery, Gr. T. Popa University of Medicine, Iasi, Romania

**Keywords:** Optic chiasm, Optic nerves, History of anatomy, Anatomists

## Abstract

**Purpose:**

The optic chiasm is an essential structure located at the skull base that stirred over time the curiosity of anatomists, who became more and more interested in its structure and function. Through centuries, the optic chiasm was viewed as a vessel crossing, a way of transporting tears secreted by the brain to the eye, integrating images, or responsible for coordinated eye movements. The paper aims to overview the history of understanding the optic chiasm from the beginnings of antiquity to the twentieth century.

**Methods:**

We reviewed the literature and studied all the historical sources on optic chiasm and eyes in the works of ancient, medieval, Renaissance authors, and the seventeenth to nineteenth century works.

**Results:**

The optic chiasm is a structure that fascinated ancient anatomists and made them develop various theories on its function. In terms of function, the optic chiasm had a history based more on speculation, the seventeenth century bringing its first understanding and reaching the peak in the nineteenth century with the understanding of the anatomical structure of the chiasm and its role in the visual process.

**Conclusion:**

The history of the optic chiasm is a fascinating time travel displaying the conceptual transformations that have been made in anatomy and medicine by our forerunners.

## Introduction

The optic chiasm is the crossroad of the visual sensory system, containing some 2.4 millions afferent axons, and it is also the conjunction of four major medical disciplines: neurosurgery, ophthalmology, neurology and endocrinology [20]. Pathological disturbance of vision stirred over the time the curiosity of the scientists and doctors, who tried to discover the mistery of optic chiasm and eyes.

### Theories about optic chiasm in antiquity

In antiquity, the father of Medicine, Hippocrates of Kos (ca. 460–370 BC) approximated the function of chiasm and optic nerves, in the period of the “Golden” Age of Greece [48]. Noticing that blows to the eyebrow could lead to blindness, Hippocrates thought that these could be involved in vision [55]. He also described the first case of traumatic optic nerve damage after craniofacial trauma: *dimness of vision occurs in injuries to the brow and in those placed slightly above. It is less noticeable the more recent the wound but as the scar becomes old so the dimness increases* [5]. Besides the involvement of chiasm and optic nerves, he also considered that these structures could also have the role of vessels transporting the waste products of the brain flowed down into the eyes, but also the tears which he believed the product of brain secretion [28, 43].

As in Ancient Greece dissections were uncommon and unacceptable due to superstitions related to the violation of human, Hippocrates could not verify his medical theories based mainly on observations [6]. Instead, this information on *nerve channels* were “proven” later by Herophilus (ca. 330–260 BC) and Erasistratus (ca. 330–255 BC), who had a deep respect for Hippocrates and his work. Professors in Alexandria in the Hellenistic Period (323–212), at the peak of Greek science, the two scholars are considered to be first human anatomists who dissected humans and animals, being known for their documented anatomy that they taught [19, 35, 52].

Although not as known as his forerunner Hippocrates, the anatomist and surgeon Rufus of Ephesus (80–150 AD) was also interested in the neuroanatomy of the sellar region, performing dissections mainly of monkey brain in the ancient cultural centre of Alexandria, in a time when anatomic dissections had not been performed in Roman schools [17]. Fortunately, many of his manuscripts had survived and had became important sources of influence and inspiration for Byzantine and medieval surgeons and had been passed over until the sixteenth century [22]. Rufus was the first who understood the anatomy of the ventricular system, including the anatomy of the third ventricle, the relations with the optic chiasm and other neighbouring structures. Moreover, he was amongst the first who described the optic chiasm and agreed with his forerunners recognising its involvement in vision [22, 29].

The information on the optic chiasm of Hippocrates and later of the anatomists from Alexandria had been taken over enthusiastically by the Roman physician Galenus of Pergamon (129—ca. 200/216 AD). The last great physician of the antiquity, Galenus lived almost half a millennium after Hippocrates and saw himself as his successor. In a time when human dissections were prohibited, his studies in anatomy were based on dissections of various animals especially monkeys but the studies on the visual system were made mainly on the eyes of freshly sacrificed oxen [72]. Galenus also observed this anatomical structure *criss-crossed* at the base of the brain that he called *chiasm* after its resemblance with the Greek letter *chi* (*χ*) [19, 47]. Nevertheless, he believed that optic nerves did not cross in chiasm and remained on their side and that there existed communication amongst them in the chiasm [19].

In his book *De Usu Partium Corporis Humani* (*On the Usefulness of the Parts of the Body*), Galenus tried to build a theory of vision in which the optic chiasm was involved by localising in it the mechanism underlying binocular vision [40]. His observations that the experimental pressure in the anterior part of the lateral ventricles produced blindness [24] made him believe that the origin visual pathways were localised in the anterior ventricle of the brain. Chiasm was seen by him a place in which *spiritus animalis* released by the cerebral ventricles reunited to further move along the hollow tubes of the optic nerves towards the eyes [19, 71].

After the death of Galenus around 200/216 AD, the anatomic dissections had been prohibited both in Europe and in Islamic countries for almost 1000 years; the dissections restarted in the thirteenth century in Italy for forensic purposes [59]. Right after the fall of Alexandria in 642 AD, the knowledge on optic chiasm had been spread also to the Arab world where these had been kept by the Arab physicians until the European Dark Ages so as at the beginning of the fourteenth century, these were found in the curricula of the European medical schools. The spreading of Galenus’ theories on the optic chiasm and the eye in the Arab world was made possible mainly due to Christian translators in ecclesiastical libraries and “court academies” of Mesopotamia, Syria or Egypt [52]. In this regard, a key role was played by Hunain ibn Ishaq (ca. 809–ca. 873) a famous scholar, physician, philosopher and translator, also named *the sheik of translators*.

### The contributions of Arab physicians to optic chiasm

The medieval Arabic science did not change significantly the ideas of Galenus and his predecessors on the role of optic chiasm. Instead, opposed to his vision, the Persian scholar, physician and philosopher Abu Bakr Muhammad ibn Zakariya al-Rhazi known as Rhazes (ca. 865–925) [77] made an assumption that a total decussation of the optic nerves existed in the optic chiasm [34]. The same thing was also suggested by Avicenna (ca. 980–1037), whom Newman viewed as the first who described the optic nerve crossing to the controlateral side [45]. In terms of other functions of the chiasm, optic nerves and tear transporters, Avicenna also accepted the theory of the ancient scholars without explaining though how this process was put into practice [1].

In the same period, in Persia, the physician Esmail Jorjani (1042–1137) was the first who identified in the optic chiasm the crossing of fibres, and the physiological significance of this was viewed as the earliest description of this phenomenon [57]. Also, Jorjani sustained that this confluence, by uniting the information from the two eyes, prevents diplopia. As Avicenna, Jorjani was aware of the involvement of optic chiasm in the binocular vision [57].

### New theories on optic chiasm at the beginning of the thirteenth century

**Fig. 1:**
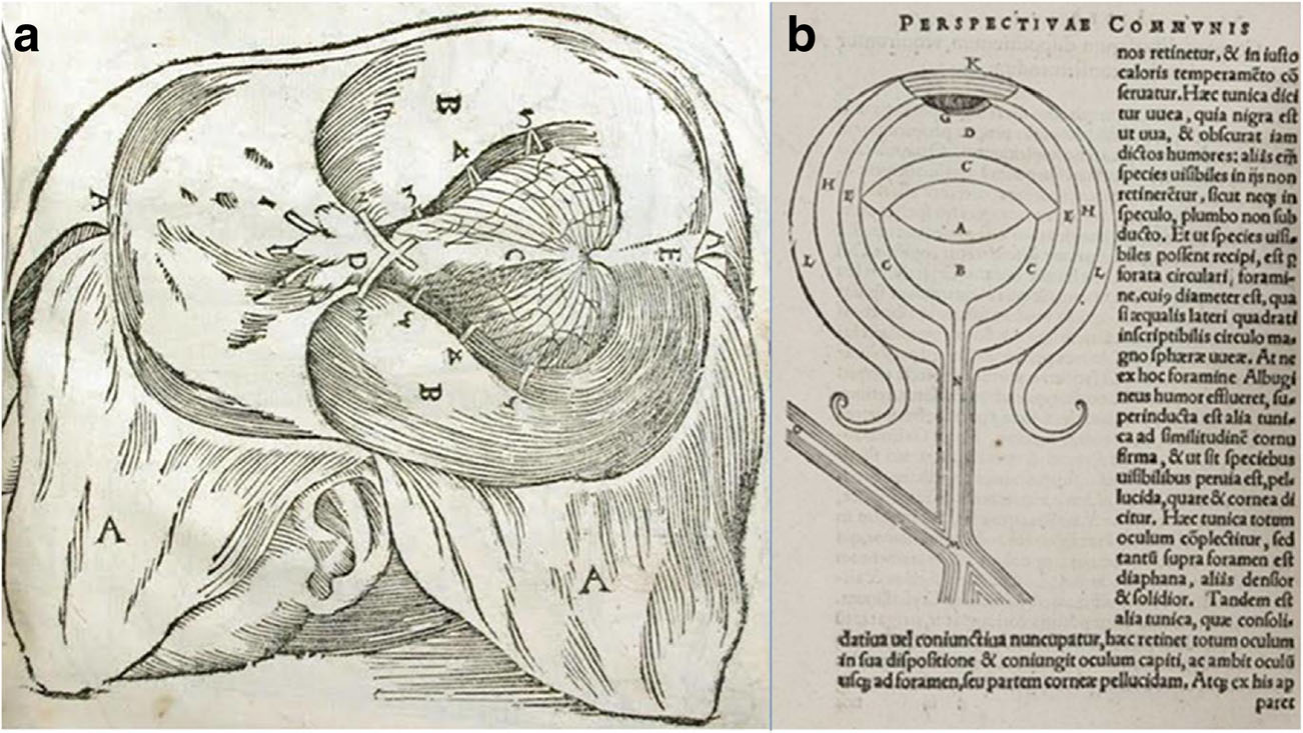
The representation of the optic chiasm by Mondino de Luzzi in *Anathomia corporis humani* (1316) (**a**) and by John Peckham in *Optics (Perspectiva communis)* (**b**) (public domain)

Together with the restarting of dissections in the thirteenth century, the first European manual of anatomy appeared, *Anathomia corporis humani*, written by the anatomist and professor of surgery Mondino de Luzzi (ca. 1270–1326). Considered to be the first modern manual of dissection and an anatomical text, *Anathomia corporis humani* was written in 1316 and printed for the first time in 1478 [9, 12, 56]. Moreover, he summarised the anatomy of his forerunners and reintroduced human dissection. Regarding the optic chiasm, he called it *common station for optic nerves* [60]. In his book, in the seventh illustration, Mondino showed optic nerves labelled by *D* that came from the forward ventricles, joined together to form the chiasm and extend as to enter into eyes (Fig. 1). In his vision, if one eye was closed, the whole *spirit* was transferred to the other eye [12].

Also in the thirteenth century, the optic chiasm was also seen as the seat of the *Sensus communis* [15]. So, the Polish theologian and scholar Witelo (ca. 1230–1280/1314) in his most important work, *Perspectivorum libri decem*, made a diagram of the visual system. In this diagram, the images “produced” in the two eyes coincided in the optic chiasm. The treatise of Witelo had been used and inspired other scholars, such as Johann Müller, Johannes Kepler, Nicholas Copernicus and Leonardo da Vinci [11].

Also in the same period, Archbishop of Canterbury John Peckham (ca. 1230–1292) had also shown his interest for optics being influenced by his contemporary, Roger Bacon called *doctor mirabilis* and one of the greatest philosophers of the early Middle Ages. In his book *Optics*, Peckham drew a schematic illustration of the optic chiasm as a crossing of the optic nerves (Fig. 1). *Optics* had been the most popular book for the next centuries on this subject so that it was called *Perspectiva communis* as it was used everywhere [15].

### Leonardo da Vinci’s view on the optic chiasm

**Fig. 2:**
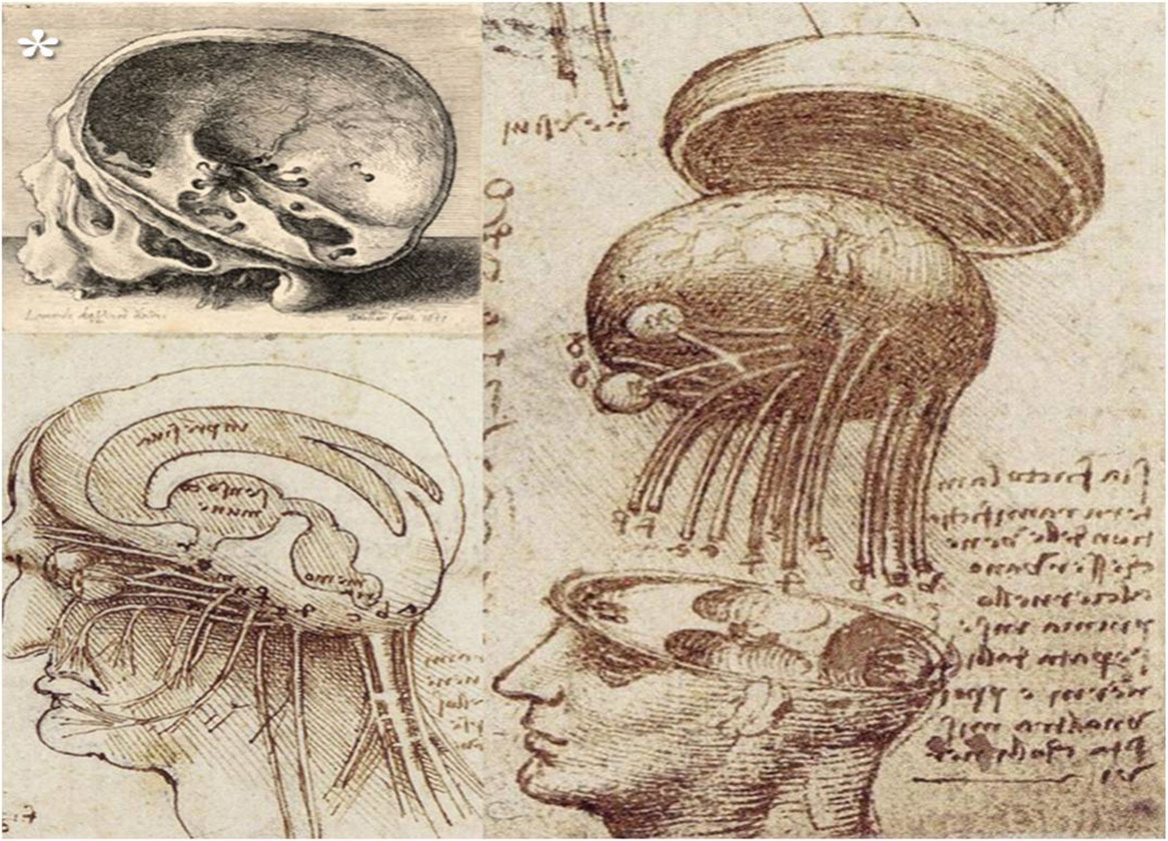
The optic chiasm in Da Vinci’s vision (*asterisk* indicates the reprint of Leonardo da Vinci of *Wenceslaus Hollar*, 1607–1677) (public domain)

After almost 1000 years, anatomic dissections had started to be allowed in the thirteenth century, together with the growth of the medical schools in Europe, initially in Italy for forensic purposes and later for the study of medicine. During that period of anatomical knowledge flourishing, Leonardo da Vinci (1452–1519) was born, considered to be the first great medical illustrator [38] and one of the earliest contributors to the history of anatomy [62]. He was permitted to dissect human corpses at the *Hospital of Santa Maria Nuova* in Florence and later in hospitals in Rome or Milan [44], and he completed approximately 30 dissections during his lifetime [4]. So, da Vinci succeeded to make the first anatomical diagrams of cranial nerves, including the optic chiasm, making the three-dimensional construction of the brain, as well as the first wax casting of the ventricular system with the earliest anatomical 3-D reconstructions of these cavities [22, 23, 41]. Through his diagrams of the brain and the eye, including the optic chiasm that he illustrated correctly (Fig. 2), da Vinci is viewed as the person who stated the approximate precision of the anatomical relations with the chiasm.

Concerning the role of the optic chiasm, besides its involvement in vision, he believed that it was responsible for the conjugated movements of the eyes [29, 71]. Also, *il Maestro* believed that the seat of soul was located just above the optic chiasm, close to the anterior portion of the third ventricle. He argued this localisation based on his observations that the disturbances of this area affect the perception of the inner and outer world [13, 54]. In what regards the issue of crossing or non-crossing of the optic nerves in the optic chiasm, da Vinci did not state anything in this regard but just drew it in his anatomical illustrations [53].

### New theories on the structure of the optic chiasm in the fifteenth, sixteenth and seventeenth centuries

The fifteenth and the sixteenth centuries were dominated by the anatomists Berengario da Carpi, Bartolomeo Eustachio, Constanzo Varolio and of course Andreas Vesalius, who, back in 1543, by publishing *De Humani Corporis Fabrica*, inaugurated the beginning of the Golden Age of Italian anatomy up to 1627. Moreover, starting with the second half of the sixteenth century, the best descriptions of the origin, structure and pathways of the optic nerve and chiasm appeared.

Initially known for treatment with mercury of the *French disease* (syphilis) [32], the Italian physician Jacopo Berengario da Carpi (ca. 1460–ca. 1530) gained the reputation of being one of the most famous physicians of the sixteenth century and close to Pope Leo X. They say that this influential friendship helped da Carpi escape the punishment for violence and suspicion of vivisection that was never proven [14, 18]. Berengario was interested in anatomy and surgery; he built a bridge between medieval Galenism and Renaissance observational anatomy [7]. Da Carpi also described the optic chiasm (1521) which he named *incruciatio* or *incruciari* [60].

**Fig. 3:**
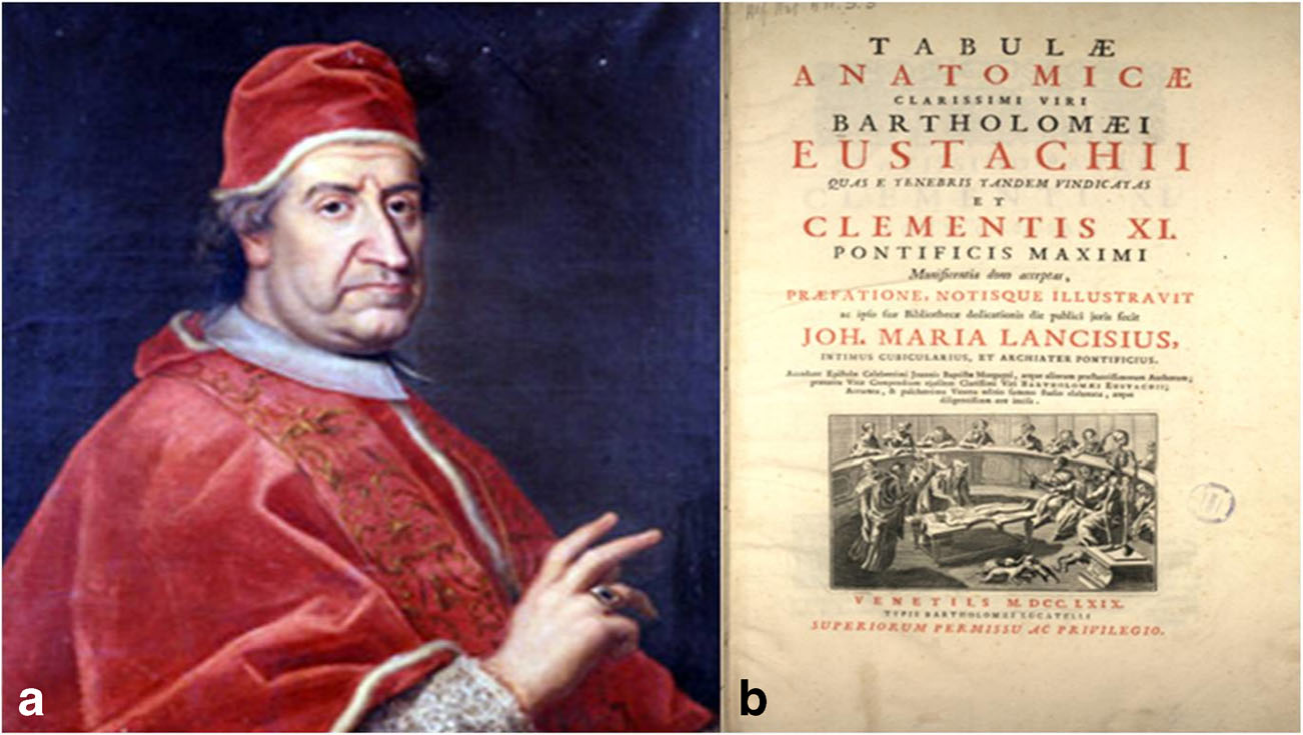
**a** Pope Clement XI (1649–1721). **b** Cover page of *Tabulae anatomicae* (1783) (public domain)

Acknowledged as being the first neuroscientist of the Renaissance who identified the correct path of optic nerves, the Italian Bartolomeo Eustachio (1524–1574) also visualised the optic chiasm that he described. He argued that it was part of the trajectory of the optic nerve and it does not project in lateral ventricles as the ancient Greeks believed and it does not project directly to the brain but first passes to the posterior part of thalamus (e.g. the lateral geniculate nuclei), although his discovery had been ignored for more than 150 years [30]. His plates provided best descriptions of the base of the brain with the visualisation of the optic chiasm. Although these were made in 1552, in *Tabulae anatomicae* (Fig. 3), they had remained unpublished and forgotten in the Vatican Library for more than 150 years until 1714 when they were discovered and published by the Pope Clement XI [2, 19] (Fig. 3).

**Fig. 4:**
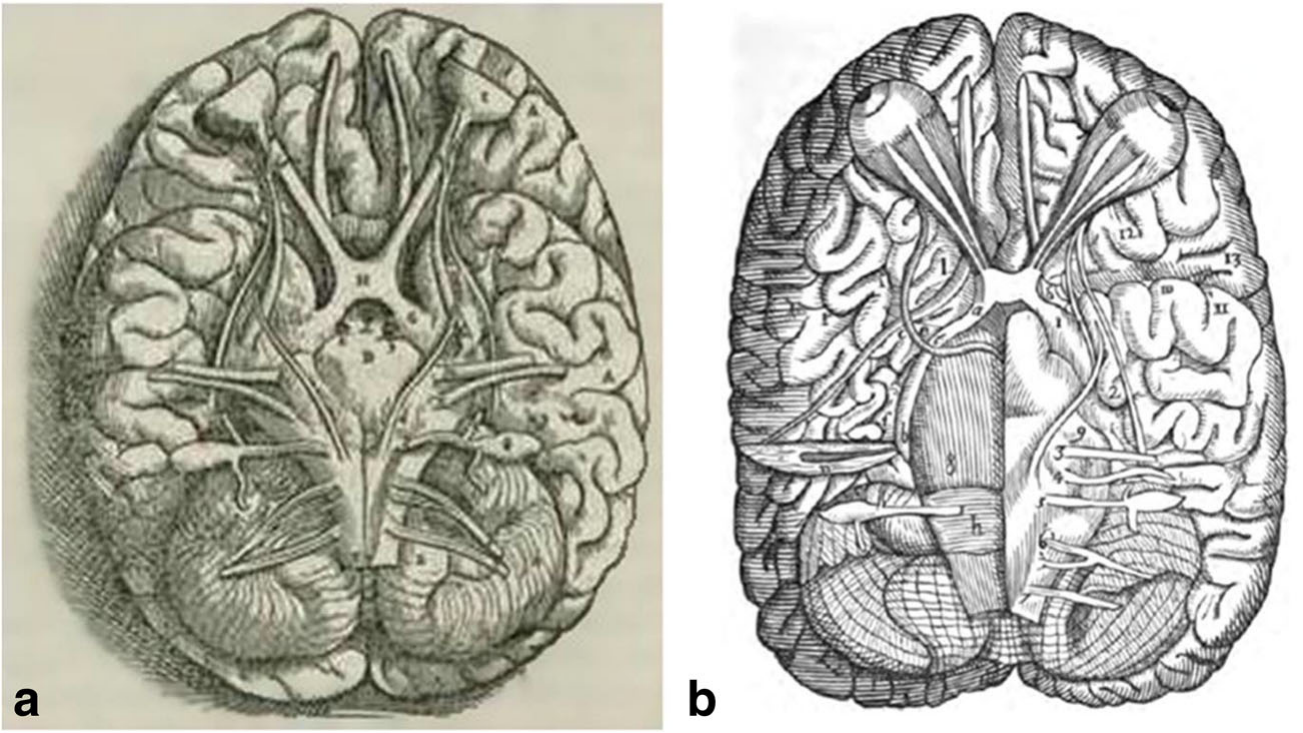
The optic chiasm in illustrations of Andreas Vesalius (**a**) and Constanzo Varolio (**b**) (public domain)

The famous professor of anatomy Andreas Vesalius (1514–1564) was one of the first who put a doubt upon the existence of the Galens’ optic nerve channel [52] in his search for *truth* in dissections of beheaded people or living animals [67, 68]. He visualised and described the anatomy of the entire sellar region [8] and drew the optic chiasm which called it in 1543 *visoriorum nervorum coitus* [60] (Fig. 4). Moreover, Vesalius described two cases of absence of optic chiasm, in which the optic nerves do not cross and remain on the same side on their entire length [53].

In 1573, the professor of anatomy and papal physician of Pope Gregory XIII (1502–1585), Constanzo Varolio (1543–1575), suggested a new method for brain dissection by its detachment from the skull base, opposed to the dissections that had been performed earlier from the upper part down [63]. The immediate result of his method had not been only the description of pons Varolio, but also a better visualisation of the chiasm. In his monograph, *De nervis opticis nonnullisque aliis, praeter communen opinionem in Humano capite observatis* (*On the optic nerves observed in the human brain and a few other particulars adverse to the common opinion*) [65] published in 1573, Varolio also reminded of the optic chiasm that he described in detail and drew it in representative illustrations (Fig. 4). Due to his new method for brain dissection, Varolio is viewed as the first anatomist who observed the entire trajectory of the optic nerve from its origin to the end in the brain [46].

Several years later, in 1595, the rector of the medical school at Montpellier André du Laurens (Laurentius) (1558–1609) introduced for the first time the term *chiasma opticum* (optic chiasm) in modern usage [51].

If the sixteenth century was predominated mainly by the description of the optic chiasm and the correct discovery of the optic nerve trajectory from the eye to the brain, starting with the seventeenth century, the scholars focused on understanding the physiology of the optic chiasm and the eye.

**Fig. 5:**
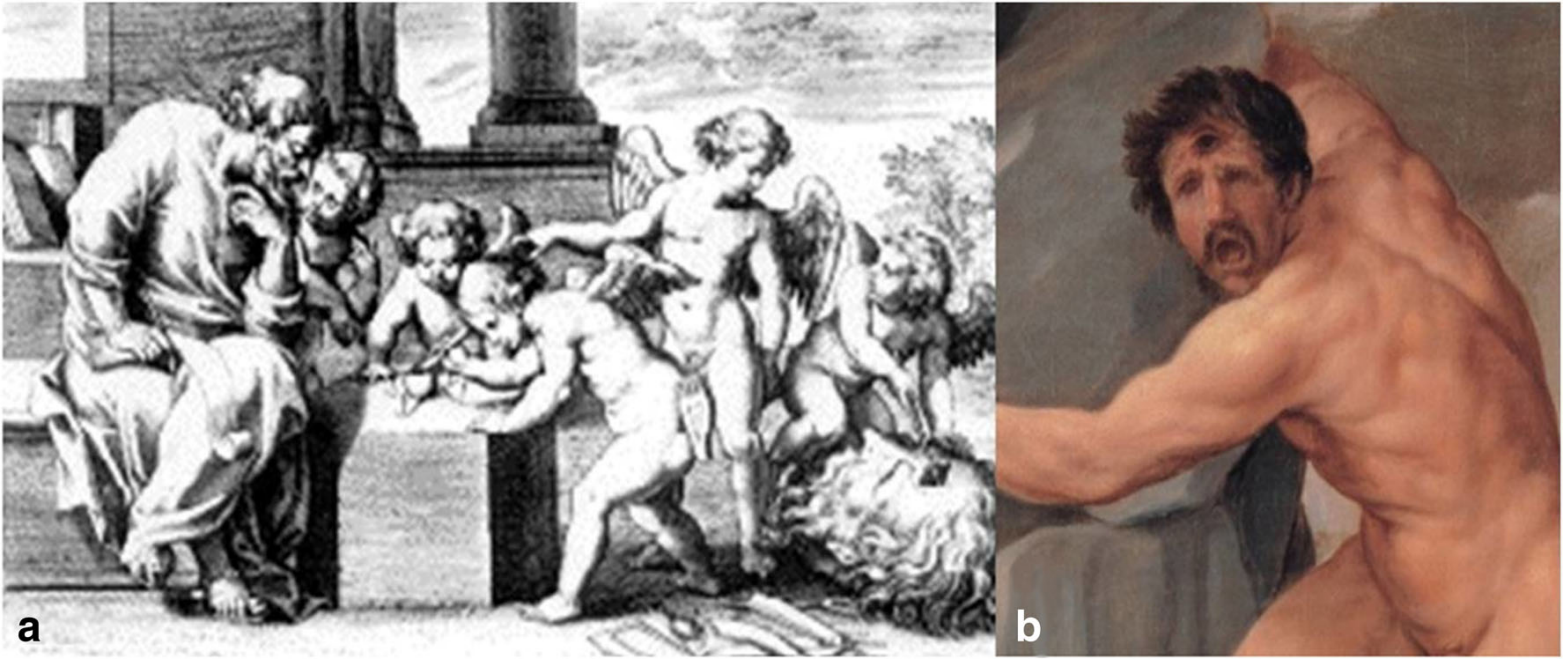
**a** Engraving by Peter Paul Rubens in the book *Opticorum Libri Sex*. **b** *Polyphemus*, Guido Reni (1639–1640), Cyclop from Greek mythology (public domain)

François d’Aguilón (1567–1617), a Jesuit monk, Belgian mathematician and physician, a passionate of optics, became known for the fact that he was the first to discuss the *stereographic process* that he named as such. Regarding the optic chiasm, Aguilón adopted Galen’s idea of the *cyclopean eye* located in the chiasm [29]. He published his work in six books that he named *Opticorum Libri Sex* [10], enriched by the engravings of the famous Flemish painter Peter Paul Rubens (1577–1640), amongst which one that represented *putti* (angels) examining eye taken from cyclops (Fig. 5). Also, he supported Vesalius theory of chiasm stating that the nerves only touch each other at the chiasm [78].

**Fig. 6:**
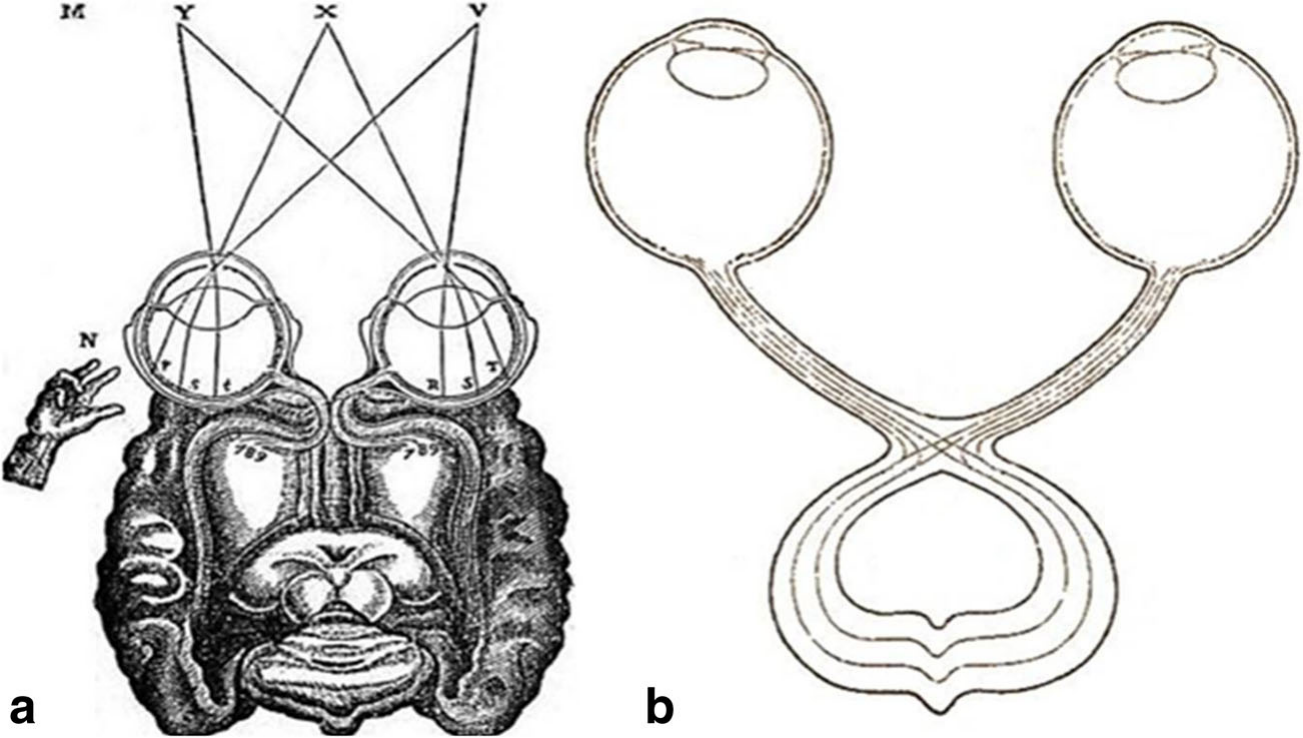
**a** The optic chiasm and the eye in the vision of René Descartes in *Dioptrique*, 1637. **b** Adaptation after schematic illustration of the optic chiasm by Isaac Newton, 1704 (public domain)

Similar to Aguilón, the French philosopher René Descartes (1596–1650) also supported the binocular vision and the absence of decussation in the optic chiasm. In the vision of Descartes, the uniting and integration of images taken over by the eyes is the process occurring in the pineal body. In one of his diagrams, in the paper *Dioptrique* (1637), Descartes made a drawing in which the ipsilateral projection of the optic nerves occurs in the brain, and then combines in the pineal body; also, in the optic chiasm, the nervous fibres were represented as being uncrossed (Fig. 6).

Starting with the second half of the seventeenth century, the neuroscientists drew their attention upon the path of the optic nerve beyond the optic chiasm. In 1664, the famous English physician Thomas Willis (1621–1675) argued that the optic chiasm represents the functional location of the convergence of the optic nerves. Also, he believed that the optic nerve fibres after passing the optic chiasm (that he called the *coalition of optic nerves*) end in optic thalami (dorsal thalamus) (a term that at that time included both striate bodies and brain stem), which represents the highest level of the visual system. Only 20 years later, in 1684, Willis was completed by the French anatomist Raymond Vieussens (1641–1716), who suggested that the optic nerves would continue to the cerebral cortex [19]. In his paper *Cerebri anatome* published in Latin in 1664 [73] with illustrations made by the famous architect Christopher Wren, Willis represented the optic chiasm accompanied by the arterial circle that bears his name [16].

### Shaping the idea of partial decussation of the optic chiasm in the eighteenth and nineteenth centuries

If until the seventeenth century, meeting of the optic nerves was controversial and based more on speculation, the first suggestion that the two nerves would partially cross in the optic chiasm was made by the mathematician and physicist of the Cambridge University, the genius Isaac Newton (1642–1727). His attention on the optic chiasm appeared on 15 March 1682, when his friend, the physician, William Briggs (1650–1704) held a lecture. He would also teach Newton later how the eyes are dissected and explain the notions of anatomy. Briggs presented a paper in front of the Royal Society of London entitled *A new theory of vision* [3], in which he supported the theories of his forerunners stating that the optic nerves do not cross in the optic chiasm. Opposed to him, Newton suggested that the two optic nerves partially cross in the optic chiasm that is responsible for the binocular convergence. In 1704, in his paper *Opticks*, Isaac Newton correctly assumed that the fibres of the optic nerve that come from the nasal half of the retina cross to the other side forming the optic chiasm, whilst the fibres from the temporal half extend in the brain of the same side (Fig. 6). Inspired more by mathematical and physical concepts, Newton’s theory will be later confirmed by the anatomists (Hannover, Joseph and C. Wenzel), physiologists (Nicati, Johannes Müller) and pathologists (Singer and Munzer, Gudden, Cramer, Jakobsohn, Bernheimer, Bechterew) [74].

With the appearance of modern hospitals in Europe, the physicians started to integrate the theoretical notions related to the function of the optic chiasm to what they encountered in practice. So, in 1718, the first director of the *Academie de Chirurgie* in Paris, the surgeon Jean Louis Petit (1674–1750), was amongst the first who recognised and described visual failure caused by the pituitary enlargement by the pressure effect on the optic chiasm [39, 49]. Several decades later after Newton’s theory, in 1723, the Germans Abraham Vater (1684–1751) and Christian Heinicke were the first who applied clinically the concept of chiasmal semidecussation of the optic nerves, explaining the phenomenon of “halved vision” [66]. Vater and Heinicke are those who described for the first time the temporary loss of vision of the homonymous hemianopsia type, probably of migrainous origin, in a medical dissertation at the University of Wittenberg in Germany. The two attributed this visual defect to partial decussation of the optic nerve reaching the same conclusion as their predecessor, Isaac Newton.

Newton was followed by other researchers of the eighteenth century with similar views, such as the British ophthalmologist “Chevalier” John Taylor (1703–1772). He made in 1738 in his book *La méchanisme ou le nouveau traité de l’anatomie du globe de l’oeil* the first accurate illustration of the semidecussation of the optic nerves in the optic chiasm [61]. The diagram was possible due to his speculation and insight as the idea of chiasm crossing did not belong to him and in his paper he did not cite the reference studies of Newton, Vater or Heinicke, although he knew well their contributions to optic chiasm.

In 1755, the German Johann Gottfried Zinn (1727–1759) also studied the optic chiasm that he described in his paper *Descriptio Anatomica Oculi Humani* [79] and that he called the *quadrangular space*. Regarding the theory of crossing, he mentioned that *optic nerves extend to the eyes of the same side and are not decussated reciprocally*, citing Galen and Vesalius in his papers [53]. Considered to be the true father of the ocular anatomy [28], Zinn was a professor at the Medical Faculty of Göttingen, where he became the director of the Botanical Garden, the reputation that made the famous botanist Carolus Linnaeus (1707–1778) to name genus *Zinnea* after him.

The end of the eighteenth century also brought the completion of the anatomy of the eye and its understanding. In 1786, in his famous anatomy and physiology treatise, the French physician and anatomist Félix Vicq d’Azyr (1748–1794) showed that the anatomical brain cuts in different planes, the fact that enabled him to show the realistic image of the retrochiasmal optic pathways, including the optic chiasm or pericrural optic tracts [69]. He also understood the function of the optic chiasm calling it the *optic commissure.* Along with the optic nerves, he examined it on the microscope in horizontal section, after it had been hardened by immersion in alcohol using the newly appeared method of the German anatomist Johann Christian Reil (1759–1813) [64]. So, d’Azyr noticed that *medullary fibres* are found on the exterior side of the optic nerve, and in the chiasm, the place of union presents a homogeneous tissue [21].

A century after Vater and Heinicke, in 1824, the English chemist and physician William Hyde Wollaston (1766–1828) reported in front of the Royal Society of London his personal experience with homonymous hemianopsia [33]. From this suffering, Wollaston deducted that chiasmal decussation is not complete, and that it involves only *adjacent halves of the two optic nerves*. Four years later, Wollaston died of brain tumour, and the autopsy report showed a tumour invading the right thalamus, *as large as a hen’s egg* [58]. After Wollaston revived the theory of chiasmal semidecussation, the idea was popularised by the German anatomist and physiologist Johannes Peter Müller (1801–1858), who demonstrated in 1826 through physiological studies that lateral fibres in the chiasm do not cross on the other side [42].

The nineteenth century and especially its beginning were marked in Europe by a period of experimental studies mainly on the nervous system. In 1835, the reputed French physiologist François Magendie (1783–1855), one of the pioneers of experimental physiology, demonstrated in Paris the crossing of optic fibres in the optic chiasm by means of experimental operations on pigeons and rabbits. Magendie noted that when he cut the rabbit’s right optic tract behind the optic chiasm, his left eye became blind, and when he cut the chiasm on the midline, bilateral blindness appeared. Moreover, Magendie observed that after the removal of one eye of a pigeon, atrophy appeared along the optic nerve and controlateral optic tract [37, 58]. Through these experiments, Magendie thought that he supported the fact that human chiasmal decussation is complete [58].

In 1856, Albrecht von Graefe (1828–1870), the pioneer of German ophthalmology published an important paper in the field of neuroophthalmology, in which he described homonymous hemianopsia, binasal hemanopia and bitemporal hemianopsia [70], that entered forever into the medical vocabulary of the world. As Jean Louis Petit, one century later, Graefe argued that bitemporal hemianopsia appear in case of tumours that develop from the skull base.

Several years later, in Italy, in 1864, the Italian neurologist and psychiatrist Andrea Verga (1811–1895), known as the first who made the first reports of clinical features of acromegaly, found by performing the necropsy of a woman the presence of pituitary tumours (e.g. pituitary adenoma) that compress the optic chiasm and erode into the sphenoid sinus causing visual disorders [36].

So, in the mid-nineteenth century, the consequences of optic chiasm compression had been well shaped. With the redefining of the optical physics of the microscope and the appearance of colouring and fixation techniques and the serial sectioning of the brain in 1800 of the German anatomist Benedikt Stilling (1810–1879), the neuroscientists of the nineteenth century could visualise and define better the structure of the optic chiasm.

The first clear experiment that demonstrated partial decussation in the optic chiasm was conducted in 1870 by the German neuroanatomist and psychiatrist Johann Bernhard von Gudden (1824–1886). In 1847, Gudden investigated the optic chiasm in rabbits using serial sectioning and secondary degeneration, reporting the existence of posterior commissure (Gudden’s commissure), located immediately in the posterior angle of the chiasm of the optic nerves. He was the first who demonstrated experimentally the partial decussation in the optic chiasm of rabbits. He reached this conclusion after he had removed one eye from infant rabbits, showing that in the adult period, the surviving optic nerve from the normal eye may be traced in two distinct bundles, one found in ipsilateral and the other in controlateral optic tract [25–27]. By fine dissection of the brain using a specialised microtome patented by von Gudden, he managed to clarify better commisural fibres of the optic chiasm and tract, fibres that were later called in his honour, *commissure of Gudden.*

**Fig. 7:**
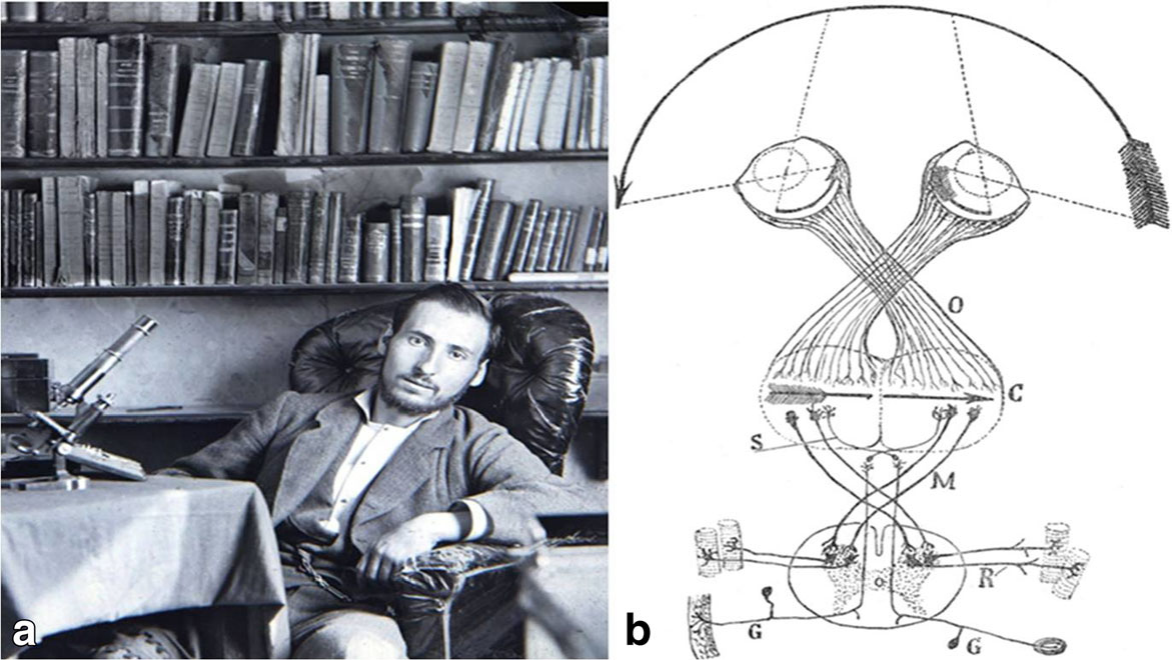
**a** Santiago Ramón y Cajal (1852–1934), one of the founders of modern neuroscience. **b** Schematic illustration of the chiasm drew by Santiago Ramón y Cajal (public domain)

During 1899–1911, Santiago Ramón y Cajal (1852–1934), a famous Spanish histologist, considered one of the founders of modern neuroscience, found that in mammalian chiasm, some axons were crossing in the chiasm and some not [75, 76]. He used Marchi and Golgi method, as well as the staining with methylene blue to show the existence of crossed and uncrossed fibres in the optic chiasm in rabbits and cats, presenting the highly representative schematic figures (Fig. 7) [27, 75]. In Cajal’s view, the brain could not operate with a disrupted a sensory space, seeing chiasm as a device for *correcting* inversion of the visual field produced by the crystalline in the eyes [27].

In the same period, the German ophthalmologist Hermann Wilbrand (1851–1935) discovered that in the optic chiasm, the nervous fibres from the lower retinal quadrants loop forward into the termination of the opposite optic nerve before passing back into the optic tact. Later, it became part of the anatomic terminology as the *Wilbrand knee* [58].

After 1950s, the information on the optic chiasm was revised and completed by Polyak, Hoyt and Luis after their experiments on macaque monkeys [31, 50].

## Conclusions

The optic chiasm is a structure that fascinated the ancient anatomists and made them develop various theories on its function. It has been studies over the centuries, and when vivisections could be performed, they reached a peak in the nineteenth century with the understanding of the anatomical structure of the chiasm and its role in the visual process. These new theories opened the path to new disciplines, such as neuroophthalmology, which flourished in the twentieth century.
